# Ambulatory Parathyroidectomy for Secondary Hyperparathyroidism at a Large Dialysis Program in Toronto: A Program Report

**DOI:** 10.1177/20543581221127937

**Published:** 2022-10-28

**Authors:** Bianka Saravana-Bawan, Bourne Lewis Auguste, Alireza Zahirieh, Karen Devon

**Affiliations:** 1 Women’s College Hospital, Toronto, ON, Canada; 2 Toronto General Hospital, ON, Canada; 3 Department of Surgery, Faculty of Medicine, University of Toronto, ON, Canada; 4 Sunnybrook Health Sciences Centre, Toronto, ON, Canada; 5 Department of Medicine, Faculty of Medicine, University of Toronto, ON, Canada

**Keywords:** hyperparathyroidism, chronic kidney disease, secondary hyperparathyroidism, parathyroidectomy, dialysis

## Abstract

**Purpose of program:**

Operative wait times for non-oncology-related procedures continue to rise in Canada, and this was further exacerbated by the COVID-19 pandemic. These challenges will remain prevalent beyond the pandemic given the limited number of acute care beds and resources required to care for patients. As a result, the need for innovative approaches to optimize the utilization of health care resources while maintaining equitable and timely access is needed. In this report, we describe the development of a collaborative ambulatory parathyroidectomy program between two centers in Toronto, allowing for more expedient surgical treatment of secondary hyperparathyroidism among patients from a large dialysis program.

**Sources of information:**

The need for expanded access to surgical care for secondary hyperparathyroidism was identified through interdepartmental communication between referring nephrologists and surgeons at Sunnybrook Health Sciences Centre and Women’s College Hospital, respectively.

**Methods:**

A multidisciplinary ambulatory parathyroidectomy planning team was formed that included nephrologists, endocrine surgeons, nurses, and patient care managers to conduct a needs assessment. It was identified that patients had long wait times, and to address that gap in care, a protocol was developed to identify suitable patients requiring treatment. The teams created a plan to coordinate patient care and transfers. A clinical tool and protocol for post-operative management of hypocalcemia was developed using a Delphi model, gathering input from many members of the care team. The Delphi process to finalize the protocol included a series of virtual meetings over a period of about 4 months with various stakeholders and included input from two departmental heads (medicine and surgery), three nephrologists, a nurse practitioner, a patient care manager, and two nurse educators. Meetings involved core members of the Nephrology Quality Improvement and Patient Safety at Sunnybrook Health Sciences Centre and finalized protocol was agreed upon by members of this group at a quarterly meeting.

**Key findings:**

In this article, we describe the development, initial deployment, and planned assessment of the ambulatory parathyroidectomy program at the Women’s College Hospital and Sunnybrook Health Sciences Centre. The primary aim of the program is to increase accessibility to parathyroidectomy for secondary hyperparathyroidism. A secondary aim was to allow patients to have streamlined care with a team that is well versed with maintenance dialysis needs and optimizing treatment of post operative hypocalcemia through standardized protocols.

**Limitations:**

Ambulatory parathyroidectomy relies on effective communication, flow, and availability of acute care beds. It is anticipated that occasionally, unexpected hospital demands, and health care disruptions may occur, which can limit efficiency of the program. We will also need to examine the cost-effectiveness of this program as it may improve access but increase costs related to the procedure. As the program is implemented, useful adaptations and policies to our protocol to help mitigate these limitations will be documented and published in our outcomes report.

**Implications:**

Ontario residents with chronic kidney disease with secondary hyperparathyroidism who have failed medical management will have increased and timely access to parathyroidectomy.

## Abrégé

**Objectif du program:**

Les temps d’attente pour les interventions non oncologiques continuent d’augmenter au Canada, une situation qui s’est aggravée avec la pandémie de COVID-19. Ce problème persistera au-delà de la pandémie en raison du nombre limité de lits en soins aigus et de ressources pour soigner les patients. Par conséquent, l’adoption d’approches novatrices pour optimiser l’utilisation des ressources en santé, tout en maintenant un accès équitable et opportun, est nécessaire. Dans ce rapport, nous décrivons l’élaboration d’un programme collaboratif de parathyroïdectomie ambulatoire entre deux centres de Toronto, lequel permettra le traitement chirurgical plus rapide de l’hyperparathyroïdie secondaire chez les patients d’un important programme de dialyse.

**Sources:**

Le besoin d’élargir l’accès aux soins chirurgicaux pour l’hyperparathyroïdie secondaire a été révélé grâce à la communication interservices entre les néphrologues traitants du Sunnybrook Health Sciences Centre et les chirurgiens de l’Hôpital Women’s College.

**Méthodologie:**

Une équipe multidisciplinaire de planification de la parathyroïdectomie ambulatoire composée de néphrologues, de chirurgiens-endocrinologues, d’infirmières et de gestionnaires de soins aux patients a été formée pour procéder à une évaluation des besoins. Il a été établi que les patients expérimentaient de longs temps d’attente et, pour combler cette lacune, un protocole a été mis au point pour identifier adéquatement les patients nécessitant un traitement. Les équipes ont créé un plan pour coordonner les soins aux patients et les transferts. Un outil clinique et un protocole de prise en charge postopératoire de l’hypocalcémie ont été mis au point à l’aide d’un modèle Delphi impliquant la participation plusieurs membres de l’équipe soignante. Le processus Delphi de finalisation du protocole a comporté, sur une période de quatre mois, une série de réunions virtuelles avec divers intervenants, ainsi que la participation de deux chefs de service (médecine et chirurgie), de trois néphrologues, d’une infirmière praticienne, d’un gestionnaire des soins aux patients et de deux formateurs en soins infirmiers. Ces rencontres ont réuni les principaux membres du *Nephrology Quality Improvement and Patient Safety at Sunnybrook Health Sciences Centre*, et ces derniers ont convenu d’un protocole finalisé lors d’une réunion trimestrielle.

**Principaux resultants:**

Cet article décrit l’élaboration, le déploiement initial et l’évaluation prévue du programme de parathyroïdectomie ambulatoire du Women’s College Hospital et du Sunnybrook Health Sciences Centre. Le principal objectif du programme est d’accroître l’accessibilité à la parathyroïdectomie pour les patients souffrant d’hyperparathyroïdie secondaire. Les autres objectifs étaient de permettre aux patients de bénéficier de soins rationalisés, grâce à une équipe qui connaît parfaitement les besoins en dialyse d’entretien, et d’optimiser le traitement de l’hypocalcémie postopératoire grâce à des protocoles normalisés.

**Limites:**

La parathyroïdectomie ambulatoire repose sur l’efficacité du flux et de la communication, et sur la disponibilité des lits en soins aigus. Il est attendu que des demandes hospitalières inattendues et des perturbations se produiront de temps à autre, ce qui pourrait limiter l’efficacité du programme. Nous devrons également examiner la rentabilité du programme, car l’amélioration de l’accès pourrait se traduire par une augmentation des coûts liés à la procédure. Au fur et à mesure de la mise en œuvre du programme, des adaptations et politiques utiles à notre protocole seront documentées et publiées dans notre rapport sur les résultats, afin d’aider à atténuer ces limites.

**Conclusion:**

Les résidents de l’Ontario atteints d’insuffisance rénale chronique et d’hyperparathyroïdie secondaire dont la prise en charge médicale a échoué auront un accès accru et opportun à la parathyroïdectomie.

## What was known before

The prevalence of chronic kidney disease (CKD) in Canada is expected to rise with an aging and growing population coupled with rising rates of diabetes, hypertension, and cardiovascular disease. The burden of CKD on patient outcomes and the overall health care system continues to rise. The cost of providing hemodialysis at a tertiary center or outpatient facility in Canada is more than $60,000 CAD per patient per year.^1^ Patients with kidney disease often require multidisciplinary care with close coordination between multiple care providers. This is equally important in the care of patients treated with KRT and secondary hyperparathyroidism (sHPT). It is common finding within this vulnerable population occurring at about 30% to 50% among prevalent patients.^2^ In addition, sHPT can present early during the course of progressive CKD with parathyroid hormone (PTH) levels becoming elevated often before abnormalities in serum calcium or phosphate are detected. This leads to abnormalities of bone mineral density being present often before dialysis therapy is initiated.^3^ Traditionally, it was believed that sHPT was limited to the skeletal system leading to extreme variations in bone turnover disease, but more contemporary data have revealed that other processes including vascular calcification, calciphylaxis, and neurologic disturbances can also occur.^3^ Therefore, in the absence of timely treatment, sHPT can result in decreased quality of life and increased mortality.^4,5^ Novel agents, specifically calcimimetics, have certainly revolutionized the care of patients and creating a shift in the treatment of sHPT away from surgical management.^6^ Despite the overwhelming success and effectiveness of calcimimetics, approximately 38% of patients will be refractory to medical treatment and need surgical intervention to control disease.^7,8^

Parathyroidectomy within this population is best performed by specialists, such as endocrine surgeons, requiring operative care in specialized centers. Unfortunately, these procedures cannot be performed as day surgeries and require close post operative monitoring, as patients are at risk for severe hypocalcemia.^9^ For this reason, although there is no American Society of Anesthesiogists (ASA) cut off for patients eligible to receive surgery at the site, parathyroidectomy for patients with sHPT were not performed at the WCH as it is an ambulatory surgical center without inpatient surgical beds.

Currently, patients are admitted postoperatively at their surgical site and dependent on levels of hypocalcemia have a length of stay which ranges from 2 days to 2 weeks. The constraints of the health care system as it relates to acute care beds has been a prevalent concern that predated the pandemic. For example, data from the Organisation for Economic Co-operation and Development^10,11^ (OECD) shows that Canada has 1.9 acute care beds for every 1000 people. These constraints were amplified with the pandemic and as a result many “lower” priority surgical cases were deferred.^12^ Surgical procedures are prioritized by pathology, with preference being given to oncology procedures.^13^ Consequently, patients with sHPT requiring parathyroidectomy are often placed on ever increasing surgical waitlists. The average wait time for parathyroidectomy in the province is up to 209 days for surgery with only 60% of patients receiving surgery within the target timeframe. Target surgical timeframe does vary based upon the designated patient priority but ranges from 56 to 182 days. Target wait times are Provincially dictated and are identical for each center. These procedures occur mostly in select academic centers due to the specialized training required. Stratified by center patient’s receiving surgery by target time frame varies across the province of Ontario with 17% at London Health Sciences Centre, 49% in Hamilton’s St. Joseph’s Health Care System, 50% at Toronto East Health Network, 67% at St. Michael’s Hospital and 89% in the Sinai Health System in Toronto.^14^

This data reflects primary, secondary, and tertiary hyperparathyroidism as a surgical indication.^14^ As such, the actual wait time for patients with secondary disease—due to their medical and surgical complexity necessitating inpatient care in specialized centers—is substantially higher with patients sometimes needing to be referred between surgeons to expedite care. The health care system is expected to do more with less and this had been exacerbated in the height of the COVID-19 pandemic. This was the primary catalyst for our program to expedite patient access to surgery and prevent some of the aforementioned complications related to uncontrolled sHPT.

Consequences of uncontrolled sHPT pose a considerable threat to patients’ overall health and quality of life. Cardiovascular risk is increased, as is the risk for fractures regardless of age, and severe and disabling pruritus can develop.^15-17^

Patients on dialysis have long-term therapeutic relationships with their care team.^18^ In adding them into the circle of care early on after surgery patient treatments can be individualized according to their needs rather than using a one size fits all approach at a center not familiar to them. Enabling continued care by the patient’s primary team, in close communication with the surgical team.

## What this adds

A coordinated care model where parathyroidectomy can be performed in an outpatient surgical setting with subsequent post operative transfer of care to the patients’ primary Nephrology team will allow for increased access to parathyroidectomy, enhanced continuity of care, and better management of postoperative hypocalcemia. The model will not only improve patient outcomes by decreasing the complications of uncontrolled hyperparathyroidism and postoperative hypocalcemia but will also result in cost savings by decreasing the overall length of patient admissions and reducing duplication of care services.

## Current State of the Program

To address this gap in patient care, a collaborative effort between the Endocrine Surgery team at the Women’s College Hospital and the Nephrology team at Sunnybrook Hospital has created an ambulatory parathyroidectomy program for patients with sHPT. Under this program, patients receive their surgical care at the Women’s College and after an appropriate period of post operative monitoring, are transferred that same day to the hospital associated with their primary dialysis site.

Patients who have failed medical management for sHPT requiring parathyroidectomy are reviewed monthly by the Nephrology and Endocrine Surgery team. From these patients, those whose co-morbidities would allow for safe surgical treatment at an ambulatory surgery site, without onsite intensive care teams, are selected based on the American Society of Anesthesiology (ASA) classification.^19^ Appropriate patients are then booked for surgery with coordination between both sites’ physicians, nursing, and transport teams.

On the day of procedure, patients are booked as the first case of the day to reduce likelihood of delay and facilitate transfer. Postprocedure, the patient is monitored at the surgical site for 6 hours and then transferred to the home dialysis site if there are no signs of bleeding. Postoperative bleeding is one of the most concerning complications that can occur after surgery in the neck due to the risk of airway compromise. For thyroid surgery, it has been established that post operative bleeding requiring intervention is most likely to occur within 6 hours postoperatively and accordingly, this timing was chosen for monitoring.^20^

**Figure 1.**
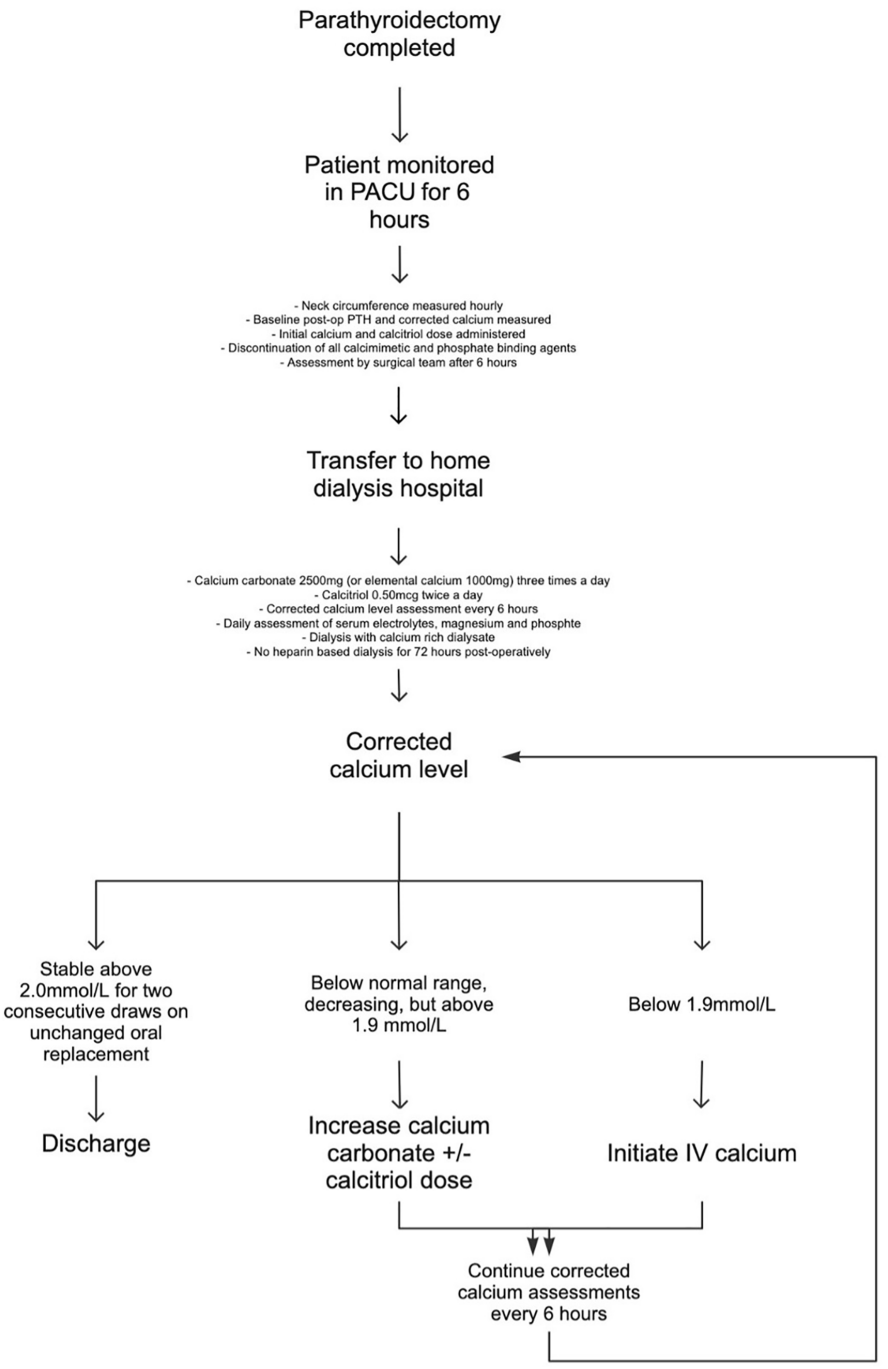
Post-parathyroidectomy care plan. *Note.* PACU = postanesthesia care unit; PTH = parathyroid hormone.

Once transferred, under the care of their primary Nephrologist, the patient is monitored, undergoes their routine dialysis, and is discharged when biochemically stable. If surgical concerns arise the surgical team from the WCH will assess the patient. Patients with sHPT are at risk of hypocalcemia postoperatively due to hungry bone syndrome.^21^ Severe hypocalcemia can be life-threatening, with symptoms of tetany, seizures, and cardiac arrhythmias.^21^ For this reason, a standardized monitoring protocol of scheduled calcium levels as well as a treatment algorithm were developed to be implemented postoperatively. It is anticipated that this protocol will lead to improved management of calcium levels, prevent episodes of symptomatic hypocalcemia and decrease requirements for IV calcium. The post operative care plan is further outlined in Figure 1.

A single case has been performed as a pilot assessment. Subtotal parathyroidectomy was completed at the WCH as an outpatient surgical procedure for a patient with sHPT on KRT on hemodialysis for 5 years. The patient was monitored, and post operative medications initiated as outlined in Figure 1. Initial PTH as assessed in the recovery unit was 12.4 from a preoperative level of 294 pmol/L and is 2.3 pmol/L at 1 month follow-up. The patient was transferred to Sunnybrook hospital, her home dialysis site, after satisfactory 6-hour postsurgical assessment by the surgical team. On post operative day 1, the patient required initiation of IV calcium replacement which was weaned as her oral calcium and calcitriol doses were increased. The patient was then discharged home with stable calcium levels on an oral replacement regimen at post operative day 7.

## Discussion: Strengths, Limitations, and Future Directions

While greatly improving patient access to care, the required co-ordination between sites can be seen as a limitation for the ambulatory parathyroidectomy program. It is anticipated, however, that while the time investment in coordinating resources may be considerable initially, this demand will decrease over time as involved teams are familiarized with the process.

Another potential limitation is that resources in terms of the home dialysis facility bed and OR time at the ambulatory surgical site are both lost if a case must be canceled. That said, careful patient selection with regards to ASA can minimize any medical causes for cancelation. Unforeseeable events can and do occur, but we anticipate that such occurrences will be rare, and we believe that the benefit of improved access to care far outweighs this risk.

The program was successfully launched in September 2021, with the performance of our first ambulatory parathyroidectomy. Future procedures are planned in the upcoming weeks and months and the team is looking to expand and include other regional hospitals to expedite the availability of parathyroidectomy for appropriate patients in these centers.

In applying the principles of quality improvement methodology, we hope that our primary outcome measure of interest is a reduction in wait time to surgery through this collaborative model. Other important measures that we will consider as part of an ongoing evaluation of this initiative which will be part of future publication is to describe balancing measures such as overall changes in parathyroidectomy rates and timing post program institution, a 30-day hospitalizations postsurgery and patient satisfaction.

## Conclusion

Our health care system like many others around the world must continue to provide optimal and timely care in the face of rising costs coupled with resource constraints. Innovative and collaborative models of care are needed to meet these current and future challenges. We describe the collaborative efforts between two large, specialized centers in Toronto, Canada, in bridging current gaps in access to timely parathyroidectomies for patients on dialysis. Programs should leverage their strengths and expertise in collaborating to meet the demands of our evolving health care system. In adopting ambulatory parathyroidectomy program, we will improve patient access to care but ongoing cost evaluation of this program to ensure sustainability and replicability will be needed.
